# Moderate-Intensity Aerobic Exercise Induces Ambulatory Hypotension in Young Adults with a Family History of Hypertension

**DOI:** 10.3390/ijerph23050602

**Published:** 2026-05-02

**Authors:** Marilene Gonçalves Queiroz, Karen Dennise Lozada Tobar, Amílcar Sabino Damazo, Lucieli Teresa Cambri

**Affiliations:** 1 Graduate Program in Health Sciences, Federal University of Mato Grosso, Cuiabá 78060-900, MT, Brazil; 2 Graduate Program in Physical Education, Federal University of Mato Grosso, Cuiabá 78060-900, MT, Brazil; 3 Graduate Program in Tropical Medicine, University of Brasília, Brasília 70910-900, DF, Brazil

**Keywords:** autonomic nervous system, blood pressure, cardiac autonomic modulation, cardiovascular response, first-degree relative, heart rate variability, hypertensive parents, parental hypertension, physical exercise

## Abstract

**Highlights:**

**Public health relevance—How does this work relate to a public health issue?**

- Hypertension is a major public health concern, and individuals with a family history of hypertension are at increased risk of developing the condition and related cardiovascular complications.
- This study evaluates a low-cost, non-pharmacological intervention with the potential to acutely reduce ambulatory blood pressure in a high-risk population.

**Public health significance—Why is this work of significance to public health?**

- A single session of moderate-intensity aerobic exercise reduced ambulatory blood pressure during both awake and sleep periods.
- These findings demonstrate immediate cardiovascular responses in non-hypertensive adults with a family history of hypertension, supporting the relevance of acute exercise effects.

**Public health implications—What are the key implications or messages for practitioners, policy makers and/or researchers in public health?**

- The findings reinforce the role of structured aerobic exercise as an accessible strategy to acutely improve ambulatory blood pressure.

**Abstract:**

This randomized crossover study aimed to evaluate the effect of a single session of aerobic exercise on 24 h ambulatory blood pressure (BP) and heart rate variability (HRV) in young adults with a family history of hypertension, FHH^+^ (participant with at least one hypertensive parent). Twenty non-hypertensive individuals (four females, sixteen males, 24.84 ± 4.15 years, 23.97 ± 3.28 kg·m^−2^) underwent a control (non-exercise) and an experimental (aerobic exercise) session in a randomized order, with a minimum interval of 72 h between them. Baseline anthropometric and metabolic parameters included body fat percentage, abdominal circumference, and blood glucose. The aerobic session consisted of 30 min cycling at 50–60% of heart rate reserve (142 ± 5 bpm; 68 ± 23 W). Twenty-four-hour BP and HRV were assessed by ambulatory monitoring. Two-way repeated-measures ANOVA showed a significant main effect of session (exercise vs. control) for systolic (*p* = 0.026, η^2^ = 0.084) and diastolic (*p* = 0.022, η^2^ = 0.088) BP, with no session × time (awake vs. asleep) interaction. For HRV indices, there were no significant (*p* > 0.05) main effects of session, nor any interaction between session and time. In summary, aerobic exercise induced 24 h ambulatory hypotension during both awake and sleep periods in non-hypertensive individuals with FHH^+^, without altering ambulatory HRV.

## 1. Introduction

Hypertension is considered a major risk factor for cardiovascular diseases, which accounted for one-third of all deaths in 2015, with an estimated 422 million prevalent cases [1]. The estimated global prevalence of hypertension in adults aged 30–79 years was 33% in 2024 [2], with fewer than half (46.5%) being aware of their condition; 36.9% receiving antihypertensive medication; and only 13.8% having controlled blood pressure (BP).

Environmental factors (i.e., obesity, sedentary lifestyle [3], stress, and salt intake) and genetic predisposition are the main determinants of hypertension. Family history (FH) is a non-modifiable risk factor for some chronic non-communicable diseases, as genetic and lifestyle (environmental and behavioral) factors are shared. Family and twin studies have suggested that 30–50% [4,5] of BP variance can be attributed to genetic inheritance, and approximately 50% to environmental factors. Based on a meta-analysis [6], our research group found that, although normotensive, young adults with FHH^+^ presented higher clinical BP and 24 h systolic BP, as well as impaired clinical heart rate variability (HRV), compared to their peers without FHH^+^. Furthermore, children of hypertensive parents have a 30–50% higher risk of developing hypertension [4]. Moreover, impaired HRV has been associated with a 32–45% increased risk of a first cardiovascular event in a population without known cardiovascular diseases [7].

A physically active lifestyle has been recognized as a non-pharmacological strategy for the prevention of hypertension. Physical exercise is effective in both the treatment and prevention of hypertension. Moderate-intensity physical training is commonly recommended by current guidelines as a first-line approach for both hypertensive and healthy individuals, due to its effectiveness in reducing BP, as well as its safety, feasibility, and high adherence [8]. Post-exercise hypotension (PEH) is at least partly mediated by changes in autonomic regulation [9]. Therefore, the combined 24 h assessment of BP and HRV provides a comprehensive understanding of the integrated cardiovascular and autonomic responses to exercise. Evidence regarding PEH in individuals with FHH^+^ remains limited. Some studies have analyzed hypotension in individuals with FHH^+^ [10,11], but none have included ambulatory analyses. Likewise, evidence regarding ambulatory PEH in normotensive individuals from the general population [12,13] remains scarce. This limitation in the literature was evident in our meta-analysis of ambulatory BP studies in individuals with FHH^+^ [6], in which only five studies included 24 h analysis, three of which reported separate daytime and nighttime results, and none assessed HRV.

Outpatient assessments using ambulatory BP and Holter monitoring have been employed to evaluate circadian rhythms by monitoring cardiac autonomic responses and BP during daily activities and in response to therapeutic interventions (e.g., physical exercise) [14]. A reduction in parasympathetic modulation during sleep following high-intensity exercise has been reported in untrained young adults [15,16]. Additionally, an increase in sympathetic modulation during sleep, reflected by higher LFnu values, has been observed in overweight/obese men after a high-intensity exercise, but not after moderate exercise [17]. Given this, it is essential to understand the alterations induced by acute exercise in cardiac autonomic modulation, particularly in individuals with higher cardiovascular risk, such as those with FHH^+^. This information may have important implications for the design of exercise programs for individuals with FHH^+^. To our knowledge, no information is available on ambulatory cardiovascular and cardiac autonomic responses to acute physical exercise in young individuals with FHH^+^. Thus, the effects of acute exercise on ambulatory cardiovascular responses in this population remain unclear. This study aimed to evaluate the effect of a single session of aerobic exercise on 24 h ambulatory BP and HRV in young non-hypertensive adults with FHH^+^. The study hypotheses were that a single session of moderate-intensity aerobic exercise would induce ambulatory PEH and changes in HRV, particularly in nocturnal HRV, in young non-hypertensive adults with FHH^+^.

## 2. Materials and Methods

Twenty individuals with FHH^+^ completed all assessments and were included in this study. Participants were recruited through social media, flyers posted on the campus of the Federal University of Mato Grosso, and lectures (online and in-person) at universities, colleges, and technical schools in the Cuiabá metropolitan area. Inclusion criteria were age between 18 and 40 years; non-hypertensive (clinic SBP/DBP < 140/90 mmHg); non-obese (BMI < 30 kg·m^−2^); and no regular engagement in structured physical exercise for at least 4 months before the study. Exclusion criteria included smoking; excessive alcohol consumption; illicit drug use; use of medications that could interfere with the assessments; presence of cardiometabolic diseases (diabetes mellitus, hypo- or hyperthyroidism); kidney disease; osteoarticular disorders limiting exercise performance; inadequate sleep patterns (sleeping < 6 or > 10 h per night); shift work; intolerance to ambulatory BP monitoring; and for the women, irregular menstrual cycle and continuous use of hormonal contraceptives.

FHH^+^ was defined as self-reported clinical diagnosis of hypertension and/or use of antihypertensive medication in at least one biological parent. FHH^+^ status was determined through structured interviews with participants, who confirmed the presence of a hypertension diagnosis in their parents, which was diagnosed before 60 years of age and present for at least one year prior to the assessments. Both parents had to be alive until at least 45 years of age. The study protocol was approved by the Ethics Committee in Human Research of the Federal University of Mato Grosso. All subjects provided written informed consent to participate in this study.

### 2.1. Study Design

**Figure 1:**
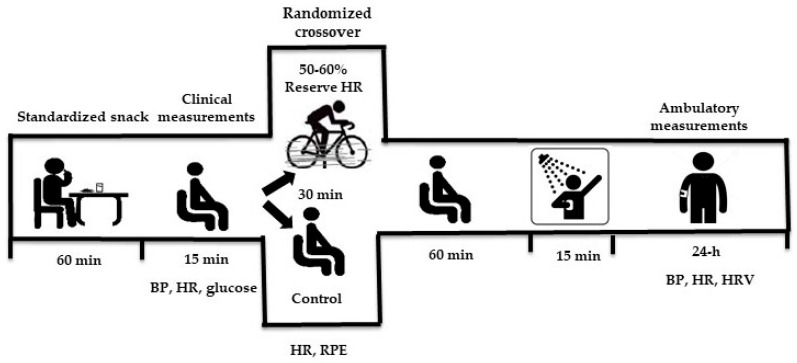
Study design. BP: blood pressure; HR: heart rate; HRV: heart rate variability; RPE: rate of perceived effort.

The study followed a randomized crossover design, in which each participant completed both the control and exercise sessions (Figure 1). Session order was determined by simple randomization using an AB/BA design, with 1:1 allocation to either the exercise/control (n = 10) or control/exercise (n = 10) sequences. All assessments were conducted between 7:00 a.m. and 1:30 p.m. in an acclimatized room. Participants attended the outpatient clinic twice; during the first visit, they completed a health history questionnaire, substance-use screening, and the International Physical Activity Questionnaire (IPAQ) [18], to confirm eligibility. During both visits, participants were instructed not to consume alcoholic and/or stimulant beverages, not to engage in intense physical exercise within the 24 h preceding the assessments, and to avoid excessive fluid intake on the day of evaluation. In addition, a standardized snack (360.7 Kcal; with 73.19% carbohydrates, 20.71% lipids, 6.10% protein) with a moderate glycemic load and glycemic index [19] was provided and consumed after an overnight fast and one hour before the start of the assessments for postprandial metabolic stabilization. The control and exercise sessions were conducted at the same time of day, with a minimum interval of 72 h between them, to avoid residual effects. During ambulatory measurements (BP and HRV), participants were instructed not to shower, do physical exercise, or consume alcoholic beverages or take any medication. They were also asked to record stressful events and/or events that deviated from their daily routine in a diary during the monitoring period, perform their usual daily activities, avoid daytime sleep, and maintain similar activities after the control and the exercise sessions, including time and duration of sleep, diet, and daily physical activity. All data were tabulated and analyzed by a researcher blinded to the experimental sessions.

### 2.2. Experimental Sessions

The control session consisted of 30 min of seated rest without any exercise. A sham cycling condition (i.e., seated on the cycle ergometer without load) was not employed because maintaining a seated position on the ergometer without pedaling may be uncomfortable and induce postural tension, potentially influencing cardiovascular and autonomic responses. The aerobic exercise session was performed on a cycle ergometer (INBRAMED, CG-04, Porto Alegre, Brazil) and consisted of a 3 min warm-up (50% of heart rate reserve), followed by 30 min of continuous aerobic exercise at 50–60% of heart rate reserve [20] using the Karvonen equation (cadence of 50–60 rpm) and rate of perceived exertion (RPE) (12–13 points) using the 20-point Borg scale [20]. This intensity is around the HRV threshold [21] and the ventilatory threshold in a similar population [22]. Heart rate (POLAR^®^, model RS800CX, Kempele, Finland) and rate of perceived exertion (RPE) were controlled every 5 min to ensure moderate intensity. Sixty minutes after each session, participants were allowed 15 min for personal hygiene needs, after which ambulatory BP and Holter monitoring were applied for a 24 h period.

### 2.3. International Physical Activity Questionnaire and Anthropometric Measurements

The IPAQ [18] was used to characterize habitual physical activity levels and confirm the absence of regular structured exercise. Body mass (OMRON Corporation HBF-514C, Kyoto, Japan) and height were assessed (SANNY^®^, stadiometer, 0.1 cm, Rio de Janeiro, Brazil) for the calculation of body mass index (BMI). Abdominal and left arm circumference were measured (CARDIOMED^®^ tape, 0.1 cm, Rio de Janeiro, Brazil) with participants standing in the orthostatic position. Body fat percentage was assessed using bioimpedance (OMRON Corporation HBF-514C, Kyoto, Japan), following the manufacturer’s recommendations. These measurements were completed in approximately 10–15 min, depending on the participant’s response time.

### 2.4. Clinical Measurements

For the clinical measurements, the participant remained seated at rest for 15 min. BP was measured using an oscillometric device (Microlife BP3T0-A, Widnau, Switzerland), validated in different populations [23]. Three consecutive measurements were obtained at one-minute intervals, and the average of the last two readings was used for analysis. When necessary, BP values were adjusted according to arm circumference and cuff size, in accordance with the criteria established by the Brazilian Guidelines for Arterial Hypertension [24].

Resting heart rate was determined based on the average of the final 5 min of the recording (POLAR^®^, model RS800CX, Kempele, Finland).

Blood glucose was assessed after BP and heart rate measurements using blood samples collected from the fingertip (Accu-Check^®^ Advantage, Basel, Switzerland).

### 2.5. Ambulatory Measurements

Ambulatory BP was monitored simultaneously with HRV using an oscillometric device (CardioMapa, Cardios^®^, São Paulo, Brazil; sampling frequency of 800 Hz). The ambulatory BP and HRV measurements presented acceptable reproducibility [25]. The ambulatory monitoring device was installed and programmed between 7:30 a.m. and 1:30 p.m. and removed after 24 h of monitoring. Monitoring of wakefulness and sleep periods was synchronized according to the sleep and wake times reported by each volunteer. Arithmetic averages for continuous 24 h, awake, and sleep periods were calculated for both BP and HRV. Participants with SBP >135, 130, or 120 mmHg and/or DBP > 85, 80, or 70 mmHg for awake, 24 h, and sleep periods, respectively, were considered to have hypertensive-range values based on ambulatory BP monitoring [26] during the control session. All protocols were conducted by the same evaluator for all participants.

### 2.6. Blood Pressure Monitoring

The cuff was properly positioned and adjusted according to the non-dominant arm circumference of each participant. Participants were instructed to keep their left arm still and relaxed alongside the body during the measurements. The device was programmed so that momentary BP values were not displayed and to obtain readings every 15 min during wakefulness and every 30 min during sleep. The assessment was only considered valid for analysis if at least 90% of the measurements were successful during both the waking and sleeping periods. The entire assessment protocol was established in accordance with the V Guidelines for Ambulatory BP Monitoring of the Brazilian Society of Cardiology [27]. The nocturnal BP dipping was calculated as dipping (%) = ((BP_awake − BP_sleep)/BP_awake) × 100. Participants were classified as dippers (≥10%) or non-dippers (<10%) [28].

### 2.7. Heart Rate and Heart Rate Variability

HRV indices were analyzed in the frequency domain using spectral analysis with the Fast Fourier Transform method. The high-frequency component (HF, 0.15–0.40 Hz), expressed in normalized units (nu), was considered an index of parasympathetic modulation, while the low-frequency component (LF, 0.04–0.15 Hz) reflects both sympathetic and parasympathetic influences, particularly mediated by baroreflex activity [29].

### 2.8. Statistical Analysis

Sample size was estimated using the G*Power software (version 3.1), assuming a repeated-measures ANOVA design (session × time), with an effect size of 0.25, α = 0.05, and power of 80%. The analysis indicated that a minimum of 19 participants would be required. Data were expressed as mean ± standard deviation. To compare 24 h variables between the exercise and control sessions, a two-way repeated-measures ANOVA was performed, considering the following factors and levels: session (exercise vs. control) and time (awake vs. sleep). Bonferroni post hoc tests were applied when significant interactions between factors were observed. The significance level was set at 5% (*p* < 0.05). Effect size for the ANOVA was calculated using partial eta squared (η^2^), with values ≥0.01 considered small, ≥0.06 medium, and ≥0.14 large [30].

## 3. Results

A total of 158 volunteers were assessed for eligibility, of whom 130 were excluded for not meeting the inclusion criteria. Twenty-eight participants were included in the study. During follow-up, six participants did not complete the second visit, and two presented issues with ambulatory analyses and declined to repeat the evaluation. Thus, 20 participants were included in the final analysis, and their clinical characteristics are presented in Table 1.

During the 30 min of physical exercise, the individuals performed ~ 68 w with a heart rate of 142 ± 5 bpm and an RPE of 12 (Table 2).

During the control session, two participants exhibited elevated ambulatory SBP across the 24 h, including wakefulness and sleep, whereas three showed elevated values only during sleep. Consequently, 25% of the sample presented hypertensive-range values based on ambulatory BP monitoring.

For SBP, there were main effects of session and time, without a significant interaction between session and time (*p* = 0.978). SBP was reduced after the exercise sessions compared to the control sessions (*p* = 0.026; −2 mmHg), both during awake and sleep periods. Additionally, SBP was lower during periods of sleep compared to awake, regardless of session (*p* < 0.01; −9 mmHg). Similarly, DBP showed main effects of session and time, without a significant interaction between session and time (*p* = 0.979). DBP was reduced after the exercise session compared to control (*p* = 0.022; −2 mmHg), both during awake and sleep periods. Additionally, DBP was lower during sleep compared to awake time, regardless of session (*p* < 0.01; −10 mm Hg); see Table 3.

Heart rate exhibited a main effect related to time only, with no significant main effect of session (*p* = 0.569) or interaction (*p* = 0.983), and was lower during sleep compared to awake periods, regardless of session (*p* < 0.01; −17 bpm). HRV indices also showed main effects of time only, with no significant main effect of session or interaction (*p* > 0.05). Specifically, LF and LF/HF were lower, while HF was higher during sleep compared to awake time, regardless of session (*p* < 0.01); see Table 3.

Acute physical exercise did not significantly modify nocturnal BP dipping, either in systolic (7.03 ± 5.02 vs. 7.28 ± 7.75%; *p* > 0.05) or diastolic BP (14.37 ± 6.47 vs. 14.41 ± 10.67%; *p* > 0.05).

## 4. Discussion

The main results of the present study are that a single session of moderate-intensity aerobic exercise induced 24 h ambulatory hypotension during both awake and sleep periods in non-hypertensive individuals with FHH^+^, without altering ambulatory HRV. Previous studies in individuals with FHH^+^ have evaluated PEH only over a short period following physical exercise [10,11]. Although participants were classified as non-hypertensive based on clinic BP, some individuals exhibited elevated values during ambulatory BP monitoring. This discrepancy highlights the importance of ambulatory BP monitoring for a more accurate assessment of BP.

While exercise appears to be more effective in hypertensive and prehypertensive individuals [31], PEH is also observed in normotensive individuals. The ambulatory BP difference after exercise is a function of initial values, such that groups with the highest baseline BP experience the greatest post-exercise ambulatory BP reductions [13]. A previous study [10] revealed that individuals with FHH^+^ who are more susceptible to hypertension are likely to derive greater benefit even after a single bout of moderate physical exercise, in terms of PEH, as compared with individuals without FHH^+^.

Although the magnitude of BP reduction was modest (~ 2 mmHg) and of limited clinical significance at the individual level, the ambulatory BP reduction observed in the present study was similar to that reported in a previous study involving normotensive young adults [12], regardless of obesity status. Notably, most of the participants exhibited a non-dipping BP pattern (nocturnal BP decrease < 10%) in both the exercise (n = 16; 70%) and the control sessions (n = 15; 75%), a profile associated with increased cardiovascular risk. While the ambulatory 24 h BP reductions after exercise are generally smaller in magnitude than those observed in clinical measurements [32], they may be more clinically relevant, as they more accurately reflect BP during activities of daily living than clinic BP measurements [33,34]. Furthermore, ambulatory BP monitoring has been shown to provide superior prognostic value for hypertension [33]. Finally, some evidence suggests that the acute BP reductions observed after a single session of aerobic exercise may be comparable in magnitude to those achieved through aerobic training [35]. However, this interpretation should be made with caution, as the present study did not assess chronic adaptations, and no inference can be made regarding long-term benefits.

It is suggested that a complex combination of BP-regulating factors, including both central and peripheral mechanisms, is responsible for PEH [36]. PEH results from persistent reductions in vascular resistance, mediated by the autonomic nervous system and vasodilator substances [9]. However, the hemodynamic determinants of PEH are controversial, due to differences in study populations and protocols. A review [37] reported that among the mechanisms proposed to explain the PEH, reduced peripheral vascular resistance was observed in 70% of studies involving continuous exercise, with 58% conducted in the morning, and participants in a sitting position during recovery, particularly in young, non-obese individuals. In addition, a study involving normotensive women with FHH^+^ [38] demonstrated that physical training reduced endothelin levels and increased nitrite/nitrate concentrations. Whether similar mechanisms are involved in the BP response to acute exercise in individuals with FHH^+^, however, remains unknown as no studies have specifically investigated these mechanisms in this population. Although PEH is at least partly mediated by changes in autonomic regulation [9], no changes were observed in ambulatory HRV. This discrepancy cannot be fully resolved with the present data, as the lack of direct measures of autonomic and hemodynamic responses limits definitive interpretations. It may be partly explained by the use of wakefulness and 24 h averages, which can mask transient autonomic responses. Furthermore, the analysis was limited to frequency-domain HRV indices, and sympathetic modulation was not directly assessed, given that the LF component reflects both parasympathetic and sympathetic influences.

Post-exercise autonomic modulation may require 24 h for vagal activity to return to baseline or even exceed pre-exercise levels [39,40]. A sustained reduction in post-exercise HRV may be associated with adverse health conditions, as it may prolong exposure to periods of increased cardiovascular risk [41]. Previous studies have shown that during wakefulness, HRV is markedly altered in the first hours following exercise [14,15]. During sleep, reductions in HRV appear to depend on exercise intensity [15,17,40,42] and duration [42]. In addition, HRV alterations during sleep may depend on the time of day at which the exercise is performed. In our study, aerobic exercise was conducted in the morning, whereas in other studies, it was performed in the afternoon or evening [15,16,42]. In the present study, moderate-intensity exercise did not alter ambulatory HRV. These findings corroborate previous studies conducted in other populations (e.g., overweight/obese [17] and sedentary healthy men [43]). It is well established that nocturnal autonomic regulation is characterized by a predominance of parasympathetic activity under normal physiological conditions [14,15,17]. These expected physiological differences between awake and sleep periods were observed in the present study, regardless of the experimental session.

The absence of significant differences in HRV outcomes may reflect limited statistical power rather than a true lack of effect. The study may have been subject to a Type II error, particularly given the inherent variability of HRV measures and the small effect sizes expected in autonomic adaptations. Accordingly, these findings should be considered inconclusive within the constraints of the present design, and any potential autonomic effects are likely small, requiring larger samples or more sensitive analytical approaches to be detected. Despite the use of 24 h recordings and separate analyses for sleep and wake periods, subtle autonomic changes may have gone undetected, as the analysis was limited to frequency-domain indices.

This study has some limitations: (a) family history was self-reported and may be subject to bias, as commonly observed in similar studies; (b) the sample consisted predominantly of men, which may limit the generalizability of the findings to women; (c) although the sample size was determined through a priori power analysis, the relatively small number of participants may limit the generalizability of the findings; (d) the exercise session was performed on a cycle ergometer between 7:30 a.m. and 1:30 p.m.; therefore, the results cannot be extrapolated to other exercise modalities or different times of day, as these factors may influence the observed findings; (e) despite both exercise and control sessions being performed in a seated position, differences in posture, muscle activation, and contextual factors between the cycle ergometer and the chair may have influenced BP and autonomic responses, which should be considered when interpreting the findings, given potential limitations in internal validity; (f) sleep–wake cycles, diet, and daily physical activity were assessed by self-report, which may introduce reporting bias and limit the control of routine consistency following the control and exercise sessions; and (g) the frequency-domain HRV indices may be influenced by several factors, including respiratory patterns, mechanical events (e.g., atrial stretch), and baroreflex sensitivity, independently of changes in cardiac autonomic modulation.

This study highlights the physiological relevance of PEH as an acute response associated with BP regulation in individuals with FHH^+^. These findings indicate that moderate aerobic exercise can elicit transient reductions in BP in this population. Additionally, although HRV is not the only factor necessary for exercise safety assessment, a similar ambulatory HRV response in both experimental sessions could represent adequate autonomic cardiac safety for moderate aerobic exercise.

Future research examining ambulatory responses to physical exercise performed at different times of the day, as well as with varying intensities and durations, in non-hypertensive individuals with FHH^+^ is warranted. In addition, longitudinal studies are needed to determine whether the acute responses observed translate into longer-term changes in BP in individuals with FHH^+^.

## 5. Conclusions

A single session of moderate-intensity aerobic exercise induced 24 h ambulatory hypotension, during both awake and sleep periods in non-hypertensive individuals with FHH^+^, without altering ambulatory HRV.

## Figures and Tables

**Table 1:** Clinical characteristics in individuals with FHH^+^.

n = 20	Mean ± Standard Deviation
Sex (Men)	80%
Age (years)	24.84 ± 4.15
Body mass (kg)	73.00 ± 12.18
Body mass index (kg·m^−2^)	23.97 ± 3.28
Abdominal circumference (cm)	83.88 ± 8.78
Body fat (%)	24.66 ± 8.64
Systolic blood pressure (mmHg)	110 ± 12
Diastolic blood pressure (mmHg)	70 ± 6
Heart rate (bpm)	76 ± 11
IPAQ (MET’s·min^−1^·week^−1^)	1165 ± 1297
Blood glucose (mg.dL^−1^)	99.10 ± 13.22

**Table 2:** Physical exercise indicators during moderate-intensity aerobic exercise.

n = 20	Mean ± Standard Deviation
Heart rate (bpm)	142 ± 5
Workload (W)	67.79 ± 23.36
Rate of perceived effort	12 ± 2

**Table 3:** Ambulatory BP and HRV indices after control and exercise session in individuals with FHH^+^.

n = 20	Time	Session				Two-ANOVA		
		Control	Exercise	All		Session	Time	Session X Time
Systolic BP (mmHg)	24 h	120 ± 9	118 ± 7	119 ± 8	*p*	0.026	<0.01	0.978
	Awake	122 ± 9	119 ± 7	121 ± 8	η^2^	0.084	0.244	0.001
	Sleep	113 ± 9	110 ± 8	112 ± 8 ^‡^				
	All	118 ± 10	116 ± 8 *					
Diastolic BP (mmHg)	24 h	69 ± 6	67 ± 5	68 ± 5	*p*	0.022	<0.01	0.979
	Awake	71 ± 6	70 ± 5	70 ± 6	η^2^	0.088	0.414	0.001
	Sleep	61 ± 6	59 ± 7	60 ± 7 ^‡^				
	All	67 ± 8	66 ± 7 *					
Heart rate (bpm)	24 h	75 ± 9	74 ± 7	74 ± 8	*p*	0.569	<0.01	0.983
	Awake	80 ± 12	80 ± 9	80 ± 11	η^2^	0.006	0.414	0.001
	Sleep	63 ± 10	63 ± 7	63 ± 8 ^‡^				
	All	73 ± 13	72 ± 11					
HF (n. u.)	24 h	31.19 ± 11.22	29.33 ± 10.43	30.26 ± 10.73	*p*	0.179	<0.01	0.977
	Awake	26.45 ± 10.94	24.68 ± 9.69	25. 56 ± 10.24	η^2^	0.031	0.268	0.001
	Sleep	42.45 ± 14.79	39.95 ± 16.63	41.20 ± 15.58 ^‡^				
	All	33.36 ± 13.98	31.32 ± 13.99					
LF (n. u.)	24 h	67.29 ± 11.19	69.16 ± 10.19	68.23 ± 10.60	*p*	0.334	<0.01	0.680
	Awake	73.39 ± 10.97	75.32 ± 9.69	74.35 ± 10.26	η^2^	0.016	0.293	0.013
	Sleep	57.71 ± 14.86	57.39 ± 14.12	57.55 ± 14.31 ^‡^				
	All	66.13 ± 13.88	67.29 ± 13.57					
LF/HF	24 h	3.56 ± 1.49	4.06 ± 2.25	3.81 ± 1.90	*p*	0.126	<0.01	0.456
	Awake	4.34 ± 1.83	4.94 ± 2.87	4.64 ± 2.40	η^2^	0.041	0.274	0.027
	Sleep	2.16 ± 1.42	2.11 ± 1.38	2.13 ± 1.38 ^‡^				
	All	3.35 ± 1.81	3.70 ± 2.51					

BP: blood pressure; η^2^: partial eta squared; HF: high-frequency component; LF: low-frequency component; η^2^: partial eta squared. * Difference between exercise vs. control (session main effect). ^‡^ Difference between awake and sleep periods (time main effect).

## Data Availability

The data that support the findings of this study are available from the corresponding author upon reasonable request.

## Abbreviations

The following abbreviations are used in this manuscript:

BMI	Body mass index
BP	blood pressure
DBP	diastolic blood pressure
HR	heart rate
HF	high-frequency component
HRV	heart rate variability
IPAQ	International Physical Activity Questionnaire
LF	low-frequency component
PEH	post-exercise hypotension
SBP	systolic blood pressure
